# Amphiphilic Acrylic Nanoparticles Containing the Poloxamer Star Bayfit® 10WF15 as Ophthalmic Drug Carriers

**DOI:** 10.3390/polym11071213

**Published:** 2019-07-19

**Authors:** Miguel Gómez-Ballesteros, Vanessa Andrés-Guerrero, Francisco Jesús Parra, Jorge Marinich, Beatriz de-las-Heras, Irene Teresa Molina-Martínez, Blanca Vázquez-Lasa, Julio San Román, Rocío Herrero-Vanrell

**Affiliations:** 1 Department of Pharmaceutics and Food Technology, Faculty of Pharmacy, Complutense University of Madrid (UCM), IdISSC, 28040 Madrid, Spain; 2 Ocular Pathology National Net (OFTARED) of the Institute of Health Carlos III, 28029 Madrid, Spain; 3 Institute of Polymer Science and Technology, ICTP-CSIC, and CIBER-BBN, 28006 Madrid, Spain; 4 Department of Pharmacology, Faculty of Pharmacy, Complutense University of Madrid (UCM), 28040 Madrid, Spain

**Keywords:** nanoparticles, glaucoma, acetazolamide, HEMA/Bayfit-MA, drug delivery, ophthalmology

## Abstract

Topical application of drops containing ocular drugs is the preferred non-invasive route to treat diseases that affect the anterior segment of the eye. However, the formulation of eye drops is a major challenge for pharmacists since the access of drugs to ocular tissues is restricted by several barriers. Acetazolamide (ACZ) is a carbonic anhydrase inhibitor used orally for the treatment of ocular hypertension in glaucoma. However, large ACZ doses are needed which results in systemic side effects. Recently, we synthesized copolymers based on 2-hydroxyethyl methacrylate (HEMA) and a functionalized three-arm poloxamer star (Bayfit-MA). The new material (HEMA/Bayfit-MA) was engineered to be transformed into nanoparticles without the use of surfactants, which represents a significant step forward in developing new ophthalmic drug delivery platforms. Acetazolamide-loaded nanocarriers (ACZ-NPs) were prepared via dialysis (224 ± 19 nm, −17.2 ± 0.4 mV). The in vitro release rate of ACZ was constant over 24 h (cumulative delivery of ACZ: 83.3 ± 8.4%). Following standard specifications, ACZ-NPs were not cytotoxic in vitro in cornea, conjunctiva, and macrophages. In normotensive rabbits, ACZ-NPs generated a significant intraocular pressure reduction compared to a conventional solution of ACZ (16.4% versus 9.6%) with the same dose of the hypotensive drug (20 µg). In comparison to previously reported studies, this formulation reduced intraocular pressure with a lower dose of ACZ. In summary, HEMA:Bayfit-MA nanoparticles may be a promising system for ocular topical treatments, showing an enhanced ocular bioavailability of ACZ after a single instillation on the ocular surface.

## 1. Introduction

Glaucoma is the leading cause of global irreversible blindness. It is estimated that 60.5 million people were affected by primary open-angle glaucoma and primary angle-closure glaucoma worldwide in 2010 [1]. This progressive optic neuropathy leads to loss of retinal ganglion cells and, by default, their axons. In this neurodegenerative disorder, there is normally a constant increase of intraocular pressure (IOP > 21 mm Hg) that damages of the optic nerve, causing an ocular neuropathy and irreversible vision loss [2]. Therefore, topical ocular hypotensive agents have become mainstream components for the management of glaucoma.

Acetazolamide (ACZ) is a carbonic anhydrase inhibitor used orally to reduce IOP in glaucoma. To obtain the desired pharmacological effect, high doses of ACZ have to be administered, commonly associated with systemic side effects, presenting a poor tolerance in most cases [3]. The deleterious systemic side effects of this drug can be avoided if it is administered topically on the eye. Local application of drugs offers several advantages, such as the possibility of lowering the dose of drug in formulations, a faster onset of action, and a marked reduction in side effects with improved patient compliance. However, ACZ is a compound with poor aqueous solubility (0.7–0.8 mg/mL) and low corneal permeability (4.1 × 10^−6^ cm/s) [3]. These circumstances limit not only the preparation of conventional ACZ eye drops, but also the ocular bioavailability of the drug [4]. One of the most employed strategies to enhance corneal drug absorption, as well as to limit non-desired systemic absorption of drugs, is the use of polymeric particulate systems [5,6,7,8].

Topical ophthalmic formulations are the most employed option for non-invasive treatment of pathologies that affect the ocular surface. However drugs’ bioavailability by this route is restricted by the short residence time of formulations, drainage, tear turnover, dilution by lacrimation, low corneal permeability, and the conjunctival absorption of the drug and scleral transport [9]. This poor ocular bioavailability can be overcome by several therapeutic approaches, such as the use of nanosystems, which are directed towards the increase of the retention time of drugs on the ocular surface and, in some cases, to enhance the corneal transport, reducing the number of applications per day and increasing the patients’ compliance [7].

Polymeric particulate systems have been widely studied in the pharmaceutical and medical fields as drug delivery systems [10]. In the ocular field, nanoparticles (NPs) have shown the capacity to deliver drugs to specific target sites, providing alternative solutions in the therapy of many ocular diseases [11]. The instantaneous formation of particles via nanoprecipitation has been attributed to interfacial interactions between liquid phases, with water essentially displacing the organic solvent, known as the Marangoni effect [12]. In nanotechnology, surfactants play major roles in the formation of nanoparticles by preventing coalescence during the preparation method, increasing stability. However, the potential toxicity and possible interactions with other formulation ingredients limit the use of surfactants in ophthalmic formulations [13].

Recently, we synthesized copolymers based on 2-hydroxyethyl methacrylate (HEMA) and a functionalized three-arm poloxamer star (Bayfit® 10WF15) (Bayfit-MA) which have an amphiphilic character, suggesting that the proposed systems could act as nanoparticulate carriers in ocular drug delivery. These novel amphiphilic materials offer the possibility to prepare nanoparticles without surfactants, representing a significant step forward in developing novel drug delivery platforms. Random copolymers were synthesized conflating amphiphilic segments of the commercial poloxamer Bayfit® 10WF15 copolymer (which belongs to a family of star PPO-b-PEO), mainly formed by 3-arm star block copolymers, and hydrophilic segments of poly(2-hydroxyethyl methacrylate) (PHEMA) arbitrarily distributed in copolymeric systems. In a previous study, we demonstrated that HEMA/Bayfit-MA copolymers did not significantly vary cellular viability, reflecting a good cytocompatibility in vitro [14]. The amphiphilic character of these novel materials suggest that the proposed systems could have application as nanoparticulate carriers in ocular drug delivery [14].

Consequently, the objective of the present study was to formulate and optimize HEMA/Bayfit-MA nanoparticles to be used in ophthalmic topical applications. The ability of these polymers to encapsulate hydrophobic hypotensive drugs with poor topical bioavailability, such as ACZ, has also been evaluated. Once prepared, nanoparticles were characterized with the analysis of particle size distribution, zeta potential, and morphology by scanning electron microscopy (SEM) and transmission electron microscopy (TEM). The final formulation was characterized in terms of osmolarity and pH. In vitro tolerability in human corneal–limbal epithelial cells (HCLE), normal human conjunctival cells (NHCs), and macrophages (RAW 264.7) was performed. This work also included in vivo tolerance and efficacy (IOP reduction) studies after the topical administration of the designed formulation in rabbit eyes.

## 2. Materials and Methods

### 2.1. Materials

Acetazolamide (ACZ) and polyethylene glycol (PEG8000) were purchased from Sigma–Aldrich (Madrid, Spain). Dimethylsulfoxide (DMSO), sodium acetate, sodium hydroxide, and dichloromethane (DCM) were obtained from Panreac (Madrid, Spain). Acetonitrile and glacial acetic acid were purchased from Lab-Scan (Gliwice, Poland). Propylene glycol (PG) was purchased from Abaran Materias Primas S.L. (Madrid, Spain). Ultrapure water was produced in the laboratory by a Milli-Q Water Purification System (Millipore Iberica SA, Madrid, Spain).

### 2.2. Quantitation of Acetazolamide

The analyses were carried out using a Gilson HPLC instrument (Middleton, WI, USA), a 305 solvent delivery pump, a 118 UV-Vis detector and UniPointTM® controller software. The injector was equipped with a 20 μL loop 7125 Rheodyne (Waters; Mildford, MA, USA). The chromatographic separation was achieved by a reversed-phase protocol with a Tracer Excel ODSA column (25 cm × 4 mm, 5 μm particle size) (Teknokroma, Barcelona, Spain). The mobile phase was a mixture of sodium acetate (Panreac, Barcelona, Spain) and ultrapure (Milli-Q) water (1:5). The flow rate was set at 1 mL/min and the eluent was monitored at 245 nm. The method was validated with respect to linearity, accuracy, and reliability in the range of concentrations between 1 and 10 µg/mL.

### 2.3. Preparation of HEMA/Bayfit-MA Nanoparticles

Copolymers of HEMA and the methacrylic derivative of Bayfit® 10WF15 (Bayfit-MA) were prepared as previously reported [14]. Copolymers were obtained by reaction of Bayfit-MA with 2-hydroxyethyl metacrylate (HEMA), with feed compositions of HEMA/Bayfit-MA at 99:1, 95:5, and 90:10 (*w*/*w*). The co-monomers were dissolved in 1 M dioxane and the mixture was deoxygenated with N_2_ for 15 min. Azobisisobutyronitrile (15 mM) was added to the solution and the reaction medium transferred to an oven at 60 °C. In order to reach high conversion, the reaction time was set to 24 h. The reaction product was washed with hexane and dried under vacuum to a constant weight. The HEMA/Bayfit-MA 99:1, 95:5, and 90:10 (*w*/*w*) were used for the preparation of nanoparticles, which were named NP1, NP5, and NP10, respectively. In brief, the corresponding copolymer was dissolved in 150 mL of DMSO at a concentration of 2 mg/mL, by heating at 100 °C and stirring until complete dissolution. The final solution was cooled down at room temperature, inserted in a dialysis bag (Spectra/Por® dialysis membrane, MWCO 3500, Spectrum Laboratories, Iberlabo, Madrid, Spain), dialyzed against distilled water with no surfactant addition to form nanoparticles, and the DMSO was removed from the nanoparticle dispersion as well. During the following 72 h, the water was changed every 3 h. Once the process was completed, the dispersion of nanoparticles was collected from the dialysis bag. Larger particles were removed by filtration (0.8 µm–25 mm Millex®-AA syringe driven filter unit, MF-Millipore Membrane). All the experiments were conducted in triplicate.

Aqueous dispersions of nanoparticles were concentrated by evaporation with a Büchi rotavapor R205 equipped with a vacuum controller V800 and a heating bath B490 (Büchi, Barcelona, Spain). This process was carried out at 30 °C under the following conditions: 20 min at 100 psi, 5 min at 75 psi, 5 min at 50 psi, 5 min at 35 psi, and around 8 min at 15 psi, which produced a final volume of 10 mL. The osmolarities of the formulations were adjusted with PG to isotonic values.

The concentration of nanoparticles in the suspensions was determined gravimetrically. First, the suspension was aliquoted in 1mL tubes that were frozen for 12 h at −80 °C and lyophilized (Flexi-DryTM MP; FTS Systems, NY, USA). Then, the samples were weighed (Mettler AG104; Mettler Toledo, Barcelona, Spain) and stored until use.

### 2.4. Preparation of ACZ-Loaded HEMA/Bayfit-MA Nanoparticles

In order to prepare ACZ-loaded particles, the polymer HEMA/Bayfit-MA 95:5 (*w*/*w*) was first dissolved in DMSO (300 mg/150 mL) by heating at 100 °C and stirring overnight until complete dissolution. Acetazolamide was then added to the polymeric solution to get a final drug concentration of 0.8 mg/mL. This solution was then inserted in a dialysis bag (Spectra/Por® dialysis membrane, MWCO 3500; Spectrum Laboratorie, Iberlabo, Madrid, Spain) and dialyzed against an aqueous solution of ACZ at a concentration of 0.8 mg/mL to avoid the loss of ACZ from the dialysis bag due to the osmotic effect. After this process, NPs were collected as described earlier (Section 2.3). Osmolarity and pH of the final formulation were adjusted with PG and NaOH 1 M, respectively.

### 2.5. Characterization of HEMA/Bayfit-MA Nanoparticles

The morphology of nanoparticles was observed by scanning electron microscope (SEM) and transmission electron microscopy (TEM) at the National Center of Electron Microscopy (ICTS) (Complutense University of Madrid, Spain). Particle size and zeta potential were carried out by photon correlation spectroscopy (PCS, Zetatrac, Largo, FL, USA).

To evaluate the content of ACZ in the formulations, NP dispersions were frozen at −30 °C and then thawed to facilitate the polymer agglomeration. Then, NPs were separated from the supernatant after centrifugation (8500 rpm, 15 min). Drug content was evaluated in the supernatant and in the NPs. To determine the amount of drug content in the particles, the precipitate obtained after the first freeze/thaw cycle underwent a second freeze cycle, was lyophilized, and finally dissolved in a mixture of DMSO:W (4:1) to obtain a final concentration of 2 mg/mL. The amount of ACZ was determined by HPLC, as described in Section 2.2.

The osmolarity was analyzed by vapor pressure measurements with an osmometer (k-7000; Knauer, Berlin, Germany) calibrated with a solution of NaCl 400 mOsM. The determination of pH was carried out using a pH meter (GLP 22; Crison, Alella, Barcelona, Spain) equipped with an Ag/AgCl combined glass electrode (Crison 52-02; Alella, Barcelona, Spain). All measurements were made in triplicate.

### 2.6. Determination of the In Vitro Drug Release

The in vitro drug release was evaluated by the dialysis method. To that end, a precise volume of the ACZ-loaded nanoparticle’s dispersion (0.8 mL) was placed into a dialysis bag (Spectra/Por Float-A-Lyzer G2; 20,000 M_W_ cut off; Iberlabo, Madrid, Spain) located inside a flask with 50 mL of a phosphate-buffered solution isotonized with NaCl (PBS, pH 7.4). The system was maintained in a shaker at 100 rpm. The collection of release media samples (1 mL) was set at 5, 15, 30 min, once every hour over a period of 8 h, and at 24 h. The media was replaced with fresh PBS, in order to maintain a constant release volume. The amount of ACZ was analyzed by HPLC following the method described in Section 2.2. The experiment was performed in triplicate.

### 2.7. In Vitro Tolerance Studies in Culture Cells

According to the assessment of the standard ISO 10933-5:2009 (Biological Evaluation of Medical Devices), the evaluation of the presence and extent of the cytotoxic effect can be undertaken by measurements of cell damage by the MTT cytotoxicity test. In this test protocol, based on the measurement of the viability of cells via metabolic activity, yellow water-soluble MTT (3-(4,5-dimethylthiazol-2-yl)-2,5-diphenyltetrazoliumbromid), is metabolically reduced in viable cells to a blue–violet insoluble formazan. The number of viable cells correlates to the colour intensity determined by photometric measurements after dissolving the formazan in alcohol. After the exposure, the formazan formation was determined for each treatment concentration and compared to that determined in control cultures. For each treatment, the percentage inhibition of growth was calculated. A decrease in the number of living cells results in a decrease in the metabolic activity in the sample. This decrease directly correlates to the amount of blue–violet formazan formed, as monitored by the optical density at 570 nm. To calculate the reduction of viability compared to the blank, the following equation is used:

*Viability (%) = OD_570e_/OD_570b_* × 100% (1)

Where $OD_{570e}$ is the mean value of the measured optical density of the 100% extracts of the test sample and $OD_{570b}$ is the mean value of the measured optical density of the blanks. The lower the viability % value, the higher the cytotoxic potential of the test item. If viability is reduced to <70% of the blank, it has a cytotoxic potential.

In vitro tolerance assays were determined by cellular viability measurements in the following cell lines: Macrophages RAW 264.7 (ATCC, Barcelona, Spain), immortalized human corneal–limbal epithelial cells (HCLE, kindly provided by Ilene K. Gipson; Schepens Eye Research Institute, Harvard Medical School, Boston, MA, USA), and normal human conjunctiva cells (IOBA-NHC; Instituto de Oftalmobiología Aplicada, Valladolid University, Valladolid, Spain). The RAW 264.7 macrophages were maintained in RPMI 1640 medium supplemented with 10% fetal bovine serum, L-glutamine (1 mM), penicillin (100 U/mL), and streptomycin (100 μg/mL) [15]. The HCLE cell cultures were plated at 15 mL culture flasks in keratinocyte serum-free medium (SFM) supplemented with 0.5 mL CaCl_2_ 0.3 M, 1.25 mL bovine pituitary extract, and 40 µL epidermal growth factor (EGF). The IOBA-NHC cells were plated at 15 mL culture flasks in DMEM/F-12 medium supplemented with 10% calf serum, 2% penicillin–streptomicin, 2.5 µg/mL amphotericin B, 1 µg/mL bovine pancreas insulin, 0.5 µg/mL hydrocortisone, 0.1 µg/mL cholera toxin, and 0.2 ng/mL EGF. Cultures were grown at 37 °C in a 5% carbon dioxide atmosphere [16,17].

Cells were seeded into 96 well culture plates (50,000 cells/well for macrophages and 40,000 cells/well for corneal and conjunctival cells). After the cells adhered to the plates, the culture medium was removed and HEMA/Bayfit-MA NPs (99:1, 95:5 or 90:10) were added (1.9 mg/mL). Viability was set as 100% in untreated cells. The vehicle used as the positive control was benzalkonium chloride 0.005% (BAK; Sigma–Aldrich, Madrid, Spain), which is the most commonly used preservative in topical ophthalmic formulations, and is especially toxic to the ocular surface cells [18]. Cytotoxicity data were obtained from three different experiments by testing seven wells per sample. Cells were exposed to formulations for 15 min, 1 h, and 4 h. Statistical differences between two mean values were evaluated by two-tailed Student’s *t*-test. If necessary, an analysis of variance (ANOVA) was employed. Results were taken as significantly different at *p* < 0.05.

### 2.8. In Vivo Short-Term Tolerance Evaluation

Experiments were carried out in non-sedated normotensive male New Zealand rabbits (3–4 kg). Animals were kept in individual cages with food and water ad libitum under controlled cycles (12/12 h light/dark). Animal studies were approved by the Ethics Committee for Animal Research of Complutense University of Madrid. Furthermore, animal manipulations followed institutional guidelines, European Union regulations for the use of animals in research, and the ARVO (Association for Research in Vision and Ophthalmology) statement for the use of animals in ophthalmic vision research.

Each rabbit received 10 µl of one of the formulations (1.9 mg/mL) in the right eye every 30 min for 6 h. The contralateral eye received the same volume of saline solution and was used as control. Three, 6, and 24 h after the first instillation, animal discomfort and symptoms and signs in the conjunctiva, cornea, and lids were macroscopically evaluated using a modification of the scoring system established in the 2002 Organization for Economic Cooperation and Development guidelines for ocular irritation testing [19].

### 2.9. In Vivo Hypotensive Efficacy of ACZ-Loaded Nanoparticles

Intraocular pressure (IOP) was measured with a Tonovet rebound tonometer (Tiolat, Helsinki, Finland). With this technique IOP was assessed without the need for topical anesthesia. The central spline of the rebound tonometer was held in a horizontal position and the distance was kept at 4–8 mm between the tip of the filament and the cornea. The assay was performed by a well-trained researcher using a well-defined set of procedures to ensure the most accurate measurements. Rabbits were treated with care and were maintained calm for at least 3 min before IOP measurement. In the case of any sign of stress, IOP measurements were postponed for at least another 3 min [20,21]. For each eye, IOP was set at 100% with two basal readings taken 30 min before and immediately before the instillation of the formulations. Then, a single dose of the formulation (25 μL) was applied to both eyes (*n* = 20 for formulations and *n* = 10 for controls). Measurements were then performed once every hour over a period of 8 h starting at 10:00. As control, rabbits received a formulation without the hypotensive agent. As reference, rabbit eyes were instilled with a conventional solution of ACZ, which contained the same drug concentration as ACZ-loaded HEMA/Bayfit-MA nanoparticles (0.8 mg/mL). In order to reduce the number of rabbits, the same animals were used multiple times as experimental and control. Consequently, protocols included a washout period of at least 48 h between experiments (ACZ has been estimated to have a plasma half-life of about 6 h [22]).

The hypotensive activity of each formulation was evaluated in terms of intraocular pressure reduction (ΔIOP), the area under the curve (AUC, %·hour) and the maximum intraocular pressure reduction (IOP_max_).

### 2.10. Statistical Analysis

The IOP reduction was expressed as means ± standard error of the means (SEM). Statistical differences among mean values were analyzed by multivariate analysis of variance (ANOVA). When necessary, two-tailed Student’s *t*-tests were employed. Graphical analyses were carried out with Origin® Pro8 software (Originlab, Northampton, MA, USA). Results were considered statistically significant when the *p*-values were < 0.05.

## 3. Results and Discussion

### 3.1. HPLC Determinations of Acetazolamide

The HPLC method to quantify ACZ was validated with respect to linearity, accuracy, and reliability in the range of concentrations between 1 and 10 µg/mL (intercept −340.5, slope 163913, coefficient of determination 0.999). Accuracy ranged between 99.5% and 100.4%, and intra- and interday precision determined by percent coefficient of variation was less than 2% in both cases. The retention time was 5 min. The method allowed sufficient separation of the drug from the vehicle.

### 3.2. HEMA/Bayfit-MA Copolymer

**Figure 1:**
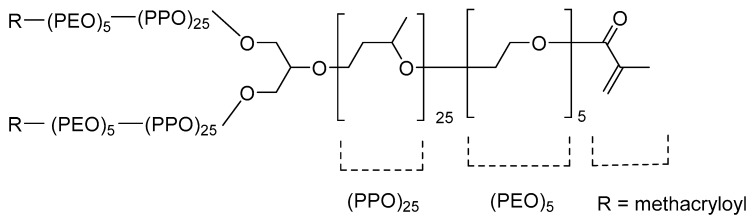
Chemical structure of the methacrylic derivative of Bayfit® 10WF15 (Bayfit-MA).

Random HEMA/Bayfit-MA copolymers obtained by free radical polymerization of HEMA and a methacrylic derivative of a commercial poloxamer, a three-arm poloxamer star, commercialized with the name of Bayfit® 10WF15, were used for the fabrication of nanoparticles. The chemical structure of the poloxamer macromonomer is shown in Figure 1. The copolymers’ compositions were HEMA:Bayfit-MA 99:1, 95:5, and 90:10 (*w*/*w*). Glass transition temperatures confirmed the random distribution of the poloxamer star along the copolymeric macromolecules (94, 93, and 95 °C, respectively). Thus, the HEMA/Bayfit-MA copolymers presented a chemical structure formed by long sequences of HEMA units (blocky sequences) randomly linked to macromonomer units of the three-arm poloxamer star.

Considering that the poloxamer stars (M_n_ = 5000 Da) provide the copolymer with a branched-like character and amphiphilic surfactant-like structure and behaviour, it was hypothesized that the copolymer macromolecules could be able to self-assemble following the usual mechanism based on the hydrophobic interaction among the hydrophobic domains of the block copolymers leading to the formation of nanoparticles in water, as has been reported for star-shaped poly(l-lactide-b-ethylene oxide) copolymers [23]. Devices with long sequences of HEMA have been proposed as potential carriers in ocular drug delivery [24]. Thus, different polymeric nanocarriers based on poloxamers are widely described in the literature [25]. One example is the use of HEMA conjunctival inserts, which can be placed in the inferior conjunctival sac of the eyes as carriers of cyclosporine A for dry eye treatment [26].

### 3.3. Characterization of Nanoparticles

#### 3.3.1. Unloaded HEMA/Bayfit-MA Nanoparticles

**Figure 2:**
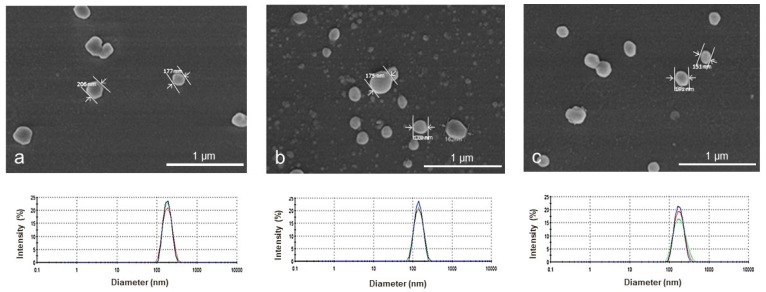
Representative scanning electron micrograph (SEM) and particle size distribution (DSL) of HEMA/Bayfit-MA NPs. Images obtained from dried (SEM) and aqueous dispersions (DSL) of NP1: (**a**) NP1 (HEMA/Bayfit-MA 99:1 *w*/*w*); (**b**) NP5 (HEMA/Bayfit-MA 95:5 *w*/*w*); and (**c**) NP10 (HEMA/Bayfit-MA 90:10 *w*/*w*).

Blank nanoparticles made using the dialysis method were analized with a scanning electron microscope (SEM) and transmission electron microscopy (TEM). Unimodal particle size distributions were observed for all nanoparticles (Figure 2) with apparent hydrodynamic diameter values of 179 ± 4, 137 ± 8, and 170 ± 3 nm for NP1, NP5, and NP10 aqueous dispersions, respectively. The average zeta potential was negative, giving values of −34.8 ± 0.1, −30.2 ± 0.9, and −28.8 ± 0.2 mV for NPs with increasing concentration of Bayfit-MA. Visualized by SEM, blank nanoparticles were spherical independent of the copolymer’s composition. Figure 2 shows some representative SEM images of HEMA/Bayfit-MA NPs.

The development of a surfactant free particulate system is highly desirable to avoid potential adverse effects on ocular mucosa. This strategy has been pursued in recent years [27,28] since Fessi et al. first developed the nanoprecipitation method to prepare poly(lactic acid) nanocapsules [12]. In fact, the dialysis method has been applied successfully without surfactant or emulsifiers in the preparation of narrow-distributed nanoparticles of block graft copolymers and other amphiphilic materials [29,30]. In this method, particles begin to form at the level of the inner surface of the dialysis membrane. After that, a layer of supersaturated solution is originated by the flux of water flowing into the dialysis tube which replaces the organic solvent. At this stage primary particles are formed. Then, the formed particles may collide due to the Brownian movements and aggregate to form secondary particles [31].

In the present work, NPs were obtained successfully without the help of surfactants, which can be attributed to the amphiphilic-like behaviour of the HEMA/Bayfit-MA copolymer system. Three different types of NPs differing in their copolymer composition (HEMA:Bayfit-MA 99:1, 95:5, and 90:10 *w*/*w*; NP1, NP5, and NP10, respectively) were obtained by dialysis of polymeric solutions in DMSO. The concentration of the initial suspensions for NP1, NP5, and NP10 was determined gravimetrically, being around 0.8 mg/mL in all cases. However, from an applicational point of view, the concentration of NPs in pharmaceutical formulations is a crucial factor that might hamper the clinical use of these types of formulations in ophthalmology. Relatively low concentrations involve the use of a high volume of particles´ dispersion in order to reach a therapeutic concentration of the drug in the target site. In order to overcome this limitation, different methods to increase the content of NPs have been proposed [14]. In this work, a high concentration of NPs was attained by evaporation in a controlled process that avoided the formation of aggregates. This process allowed us to concentrate our sample from 0.8 to 1.9 mg/mL. To assure that the formulation had enough stability and a proper size for ophthalmic topical administration, particle size distributions and zeta potential were analyzed [32,33].

#### 3.3.2. Acetazolamide-Loaded HEMA:Bayfit-MA Nanoparticles

Acetazolamide-loaded NPs were prepared from NP5 (HEMA/Bayfit-MA ratio 95:5 *w*/*w*), the co-polymer that provided good in vitro cytotoxicity results performed by means of cellular viability in HCLE cells, IOBA-NHC cells, and macrophages RAW 264.7 (as described in Section 2.7 and summarized in Section 3.4).

**Figure 3:**
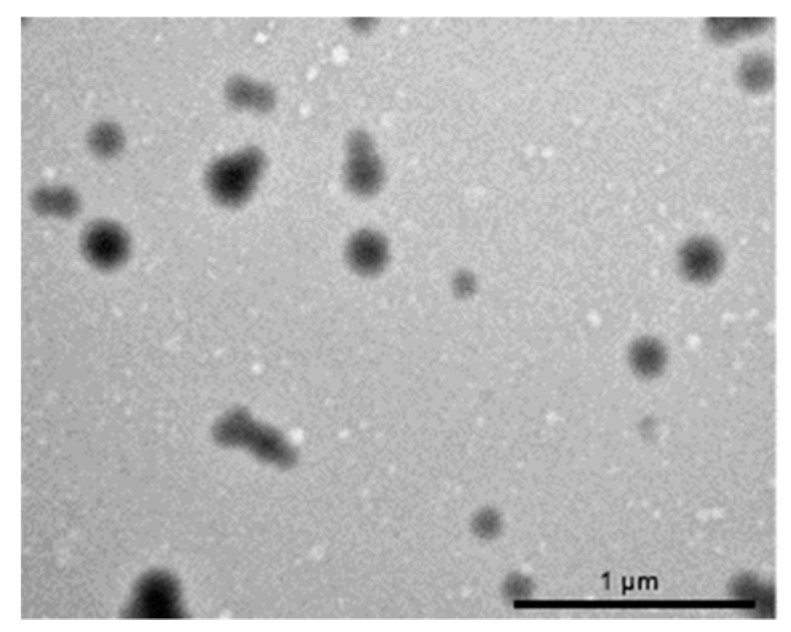
TEM images of acetazolamide-loaded HEMA/Bayfit-MA copolymer NP5 nanoparticles.

The encapsulation procedure used to prepare ACZ-loaded nanoparticles led to a yield of 21.6 ± 2.2%. Nanoparticles morphology examined by TEM revealed spherical particles (Figure 3). Particle size determinations showed a unimodal distribution with a single peak at 224 ± 19 nm. The average zeta potential was negative for ACZ-loaded NPs (−17.2 ± 0.4 mV). However, this value was less negative than the one obtained for unloaded NPs (−30.2 ± 0.9 mV), indicating that the negative charge of the polymer was partially neutralized by the positive charge of ACZ. The quantified concentration of ACZ in the final formulation was 0.8 ± 0.05 mg/mL (loading efficiency 2 µg ACZ/mg NP).

Acetazolamide-loaded nanoparticles showed a nearly neutral pH value (6.9 ± 0.2), which helped to maintain the optical properties of the eye surface, epithelial cell functions, and cellular homeostasis [34,35]. With respect to osmolarity, all of the formulations were within the range of isotonicity and acceptable for ophthalmic administration (308.3 ± 0.7 mOsm) [36].

Excipients and preservatives with surfactant properties used in eye drops are known to cause diverse ocular irritations by mechanisms such as the association with biological membranes, being able to generate changes on the surface, alterations of barrier functions, or denaturation and cell death [37,38]. In the present work, the preparation of NPs was undertaken by the dialysis method without surfactants, avoiding the adverse effects that might be associated with their use. In this method, solvents to make NPs are limited by their water miscibility. In the present work, the non-aqueous soluble DMSO was selected to prepare the nanosystems. This solvent was eliminated in the dialysis procedure as well.

### 3.4. In Vitro Release Studies

**Figure 4:**
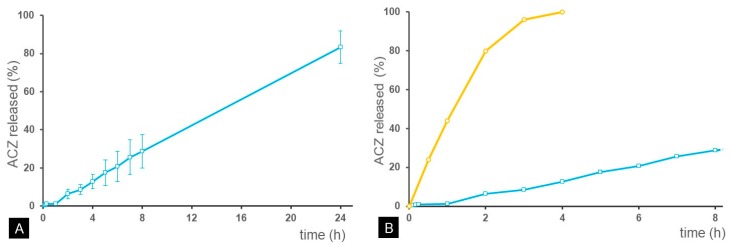
In vitro release profile of acetazolamide (ACZ): (**a**) ACZ-loaded nanoparticles dispersed in phosphate-buffered solution isotonized with NaCl (24h), (**b**) aqueous solution of ACZ (yellow) [39] versus ACZ-loaded nanoparticles (8h).

The in vitro release behaviour of ACZ-loaded NPs is summarized in the cumulative percentage of drug released, shown in Figure 4.

The release rate of ACZ was constant during the whole period. Sink conditions were maintained during the entire study. The cumulative ACZ released at 24 h was 83.3 ± 8.4%. The release study showed a zero order kinetics from the first hour of the assay and the latency period was established at 40 min. The release rate constant was calculated with the experimental data between 1 and 8 h. According to the results, the yield rate was 22.4 μg/h. In comparison with a conventional solution of ACZ [39], in which the drug diffuses completely into the release medium after 4 h (98.7 ± 6.2%), nanoparticles were able to control drug release during at least 24 h (Figure 4).

### 3.5. Viability Assays

**Figure 5:**
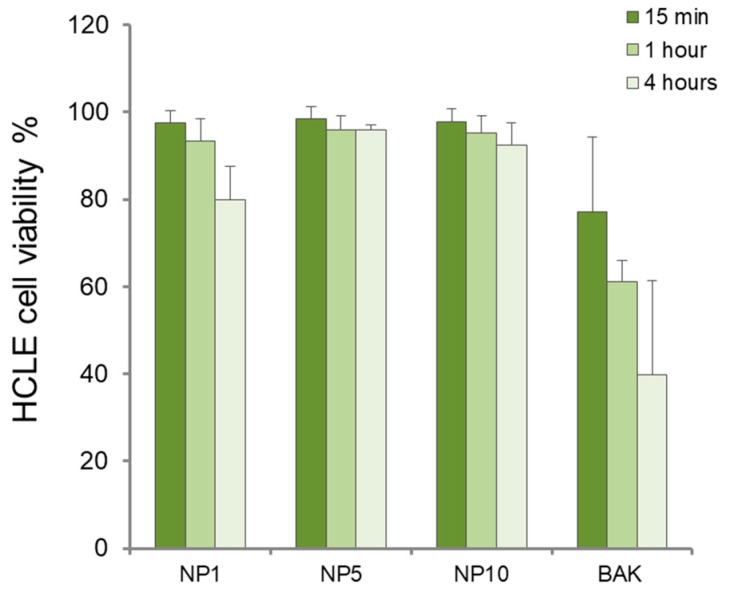
Human corneal–limbal epithelial (HCLE) cell viability in percentage (coefficient of variation, c.v.) for HEMA/Bayfit-MA copolymer nanoparticles NP1, NP5, and NP10. Benzalkonium chloride 0.005% (BAK) was used as positive control.

The cytotoxic effect of formulations composed of unloaded NP1, NP5, and NP10 at different incubation times (15 min, 1 h, and 4 h) in immortalized human corneal–limbal epithelial cells (HCLE) is shown in Figure 5. Considering that a conventional topical ophthalmic formulation is removed from the ocular surface in only 5 min, short contact times (15 min) are enough to define tolerance. However, to simulate long-term therapies and taking into account that polymers enhance the contact time of the formulations on the ocular surface, we also used contact times of 1 and 4 h in the assay. This method has previously been employed to analyze the cytotoxicity of polymeric nanosystems for topical ophthalmic administration [40,41].

Cell viability was over 80% for all samples, although the best results in terms of high cell survival (90–100%) were found for NP5 and NP10 at all incubation times. The lowest cell survival was obtained in the NP1 samples at 4 h (80.24 ± 11.6%).

**Figure 6:**
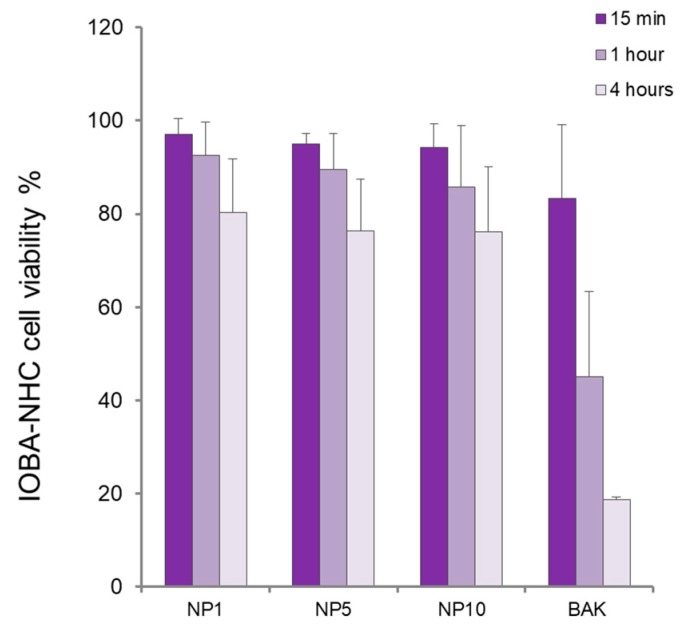
Normal human conjunctiva cell viability in percentage (coefficient of variation, c.v.) for HEMA/Bayfit-MA copolymer nanoparticles NP1, NP5, and NP10. Benzalkonium chloride 0.005% (BAK) was used as positive control.

**Figure 7:**
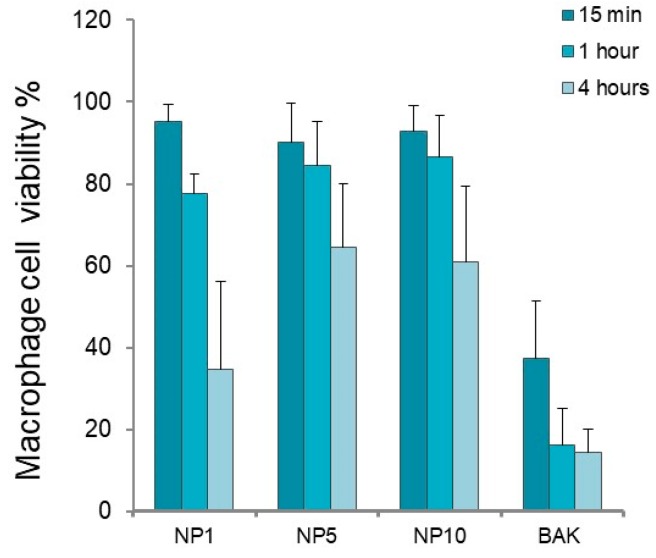
Macrophage cell viability in percentage (coefficient of variation, c.v.) for HEMA/Bayfit-MA copolymer nanoparticles NP1, NP5, and NP10. Benzalkonium chloride 0.005% (BAK) was used as positive control.

In NHC cells, viability decreased with increased incubation times for NP1, NP5, and NP10 (Figure 6). The NHC cell viability was relatively high (85–97%) after 15 min and 1 h of cell exposure to formulations. However, at 4 h, NP1 decreased cell viability to 80.2 ± 11.6%. In macrophage cells (Figure 7), after 15 min of incubation, viability was close to 100% but it nearly decreased to 80% after 1 h in all cases, indicating a good biocompatibility of the nanosystems at short and intermediate exposure times. Nevertheless, at longer exposure times, macrophages’ viability dropped to 34.6 ± 21.3% with the use of NP1, indicating a detrimental effect of NPs prepared with HEMA:Bayfit-MA 99:1 (*w*/*w*) against these type of cells, which are more sentitive under these conditions in comparison with corneal or conjunctival cell lines.

According to the assessment of the standard ISO 10933-5:2009, if viability is reduced to <70% of the blank, it has a cytotoxic potential. Given the results obtained, the only sample that significantly decreased cells viability below 70% was NP1 at 4 h of exposure (*p* = 0.0012). At these conditions, for NP5 and NP10, cell viability was not significantly different from 70% (*p* = 0.176 and *p* = 0.103, respectively). Significant differences were found when NP1 was compared with NP5 (*p* = 0.0029) and NP10 (*p* = 0.008) at 4 h in macrophages. No significant differences were obtained between NP5 and NP10 (*p* = 0.65). According to these results, a proportion of 99% of 2-hydroxyethyl metacrylate (HEMA) in the copolymer composition generated a higher reduction in cell viability, in comparison with NP5 and NP10 compositions, HEMA/Bayfit-MA of 95:5 and 90:10 (*w*/*w*), respectively. Based on these findings, in order to assure the best tolerability of the final formulation, NP5 (HEMA/Bayfit-MA ratio 95:5 *w*/*w*) and NP10 (HEMA/Bayfit-MA ratio 90:10 *w*/*w*) were selected for the in vivo short-term tolerance evaluation studies.

### 3.6. In Vivo Short-Term Tolerance Evaluation

Before performing the test, all animals in the study had normal ocular surface and corneal transparency. None had any conjunctival disorder including hyperemia or edema, eyelid swelling, or intense blinking (grade 0 according to the grading system employed, Table S1). During the study and within 24 h after the administration of NP5 or NP10, no significant signs of local reactions were observed. The topical tolerance of NP5 and NP10 was good with no clinical signs of discomfort or irritation during the follow-up period.

With the chronic use of topical ophthalmic formulations, the eye surface is continuously exposed to drugs and excipients (such as preservatives) that produce ocular surface alterations, burning or stinging sensations, which generally lead to herapeutic failure [42,43]. Thus, it is vitally important that eye drops include components that are well tolerated by the ocular surface. It is also desirable that these components enable enhanced drug bioavailability [16]. In this context, it is crucial that these new formulations provide new alternatives with good tolerability and enhanced therapeutic effects, with longer dosing intervals. According to the European Medicines Agency guidelines on non-clinical local tolerance testing of medicinal products, it is not considered essential to demonstrate the maximum tolerated dose or frank toxicity in local tolerance studies. The actual highest concentration to be used should be tested, and the dose may be adjusted by varying the frequency of administration. In the present study, formulations were applied every 30 min for 6 h, which is a much higher rate of administration in comparison with a conventional administration of hypotensive eye drops. The in vivo short-term evaluation of NP5 (HEMA/Bayfit-MA 95:5 *w*/*w*) and NP10 (HEMA/Bayfit-MA 90:10 *w*/*w*) revealed no significant differences between control and treated eyes, showing potential utility of these nanosystems as ophthalmic drug carriers. In a future work, more conclusive evidence with larger studies would be needed to assess long-term safety and tolerability. 

### 3.7. Effect of Acetazolamide-Loaded Nanoparticles on IOP in Rabbits

Considering the results obtained in the in vitro/in vivo studies (Section 3.4 and Section 3.6, respectively), both NP5 and NP10 were well tolerated, providing similar results. However, in order to diminish the number of rabbits, and in accordance with the guidelines for the use of experimental animals, the efficacy study was restricted to ACZ-loaded NP5, which contained an intermediate percentage of HEMA.

Before the experiments, we verified that the administration of unloaded NP5 did not modify IOP in the conditions of this study (data not shown). Then, the hypotensive effect produced by a single administration of a conventional solution of ACZ (reference) or ACZ-loaded NPs was evaluated (*n* = 20 for formulations and *n* = 10 for controls). Taking into consideration that the volume of the instilled formulation was 25 µL, the dose of ACZ was 20 µg in all cases.

**Figure 8:**
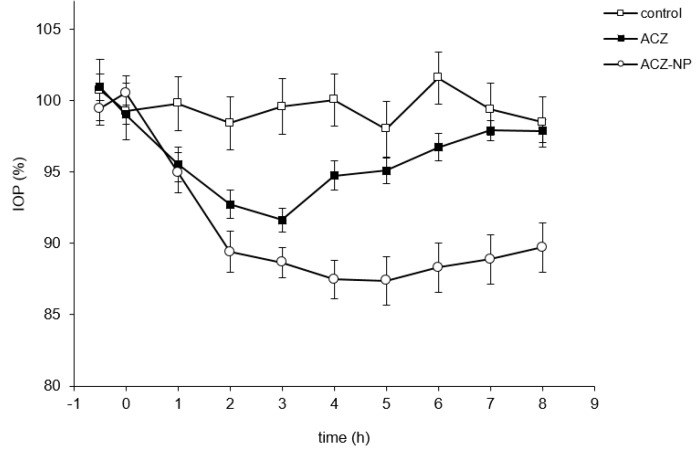
Ocular hypotensive effect of ACZ formulations. PBS was used as control. A solution of ACZ in PBS was employed as reference. Data are expressed as the mean ± SEM (*n* = 20).

The maximum percentage of IOP reduction, the time of maximum effect (t max), and the area under the ΔIOP (%)·time curve from 0 to 8 h (AUC) were calculated (Table 1). Both the ACZ-loaded nanoparticles and the aqueous solution of ACZ in PBS (reference) reduced IOP in normotensive rabbits, although the maximal effects were different for each formulation (Figure 8).

The maximum hypotensive effect was reached by ACZ-loaded NPs (16.4 ± 1%). The reference formulation achieved significantly lower values (*p* < 0.05 in all cases). The time of maximum effect was always higher for the formulation composed of the nanosystems (5 h), providing an IOP reduction that lasted longer than 8 h. Regarding AUC, ACZ-loaded NPs reached the highest value (79.8; 95% confidence interval 66.3–93.3).

Bioavailability represents the amount of drug that reaches the site of action. In this sense, the hypotensive activity can be used to establish a relative bioavailability by comparing the AUCs between the reference formulation (a conventional solution with ACZ) and the novel formulation composed of ACZ-loaded NPs. The relative bioavailability calculated from the average data was two. This value serves as an approximation of the increase in bioavailability achieved by including the ACZ in the nanoparticles.

In vivo efficacy studies in rabbits showed that the hypotensive effect of the drug was remarkably increased with the use of HEMA/Bayfit-MA nanoparticles. According to our results, the polymeric formulation generated a significantly higher IOP reduction in comparison with the conventional solution of ACZ used as reference (16.4% versus 9.6%). Other authors have tested formulations composed of ACZ that generated higher IOP reductions, with the use of liposomes [4] or niosomes [44]. However, the formulation that we developed, based on HEMA/Bayfit-MA nanoparticles, contains a lower dose of ACZ in comparison with those studies (0.5% for liposomes and 1% for niosomes versus 0.08% for the ACZ-loaded NPs developed in this work), which allows a potential reduction of undesired adverse effects associated with the drug. In future studies, it would be desirable to analyze whether the use of other components in this formulation could increase the ocular bioavailability of ACZ, prolonging the hypotensive effect and the dosing interval, or allowing further reduction of ACZ content.

Also, according to our in vivo tolerance studies, ACZ-loaded HEMA/Bayfit-MA nanoparticles did not show ocular irritancy. This fact is of great importance as the compliance of a topical treatment is linked to the absence of undesired events. In fact, in prolonged antiglaucoma treatments, the presence of adverse effects can induce lack of patient compliance and therapeutic failure [42]. As cited previously, surfactants or even the polymers employed can promote adverse effects [45]. In this work, the removal of surfactants in the preparation process was used as a technological strategy to decrease the ocular surface toxicity of the final formulation [46,47]. In fact, HEMA/Bayfit-MA 95:5 (*w*/*w*) nanoparticles were not cytotoxic according to the standard specifications in cells of the ocular strain, such as corneal and conjunctival cells, up to 4 h of contact time of cells to formulations. Unlike previously published studies [45], redness, inflammation or increased tear production were not observed immediately after the in vivo application of HEMA/Bayfit-MA nanoparticles in healthy rabbit eyes.

## 4. Conclusions

In summary, we have developed HEMA/Bayfit-MA nanoparticles which were well tolerated in vitro (cell cultures) and in vivo (rabbit eyes). This novel ACZ formulation was able to decrease the IOP in rabbit eyes and maintain the hypotensive effect in vivo during more than 8 h with a low dose of ACZ. These results suggest that HEMA/Bayfit-MA nanoparticles could be a promising ophthalmic carrier of poorly soluble drugs such as ACZ.

## Figures and Tables

**Table 1:** The IOP parameters used in the evaluation of in vivo efficacy of ACZ formulations after a single administration (25 µl, 20 µg ACZ). IOP, intraocular pressure; maximum IOP reduction (%), time of maximum effect, area under the curve from 0 to 8 h, and duration of effect (*n* = 20 eyes).

Formulation	ΔIOPmax (%)	t max (h)	AUC %·h	Effect (h)
**ACZ-loaded nanoparticles**	16.4	5	79.8	+8
**ACZ solution (reference)**	9.6	3	39.2	7

## Supplementary Materials

The following are available online at https://www.mdpi.com/2073-4360/11/7/1213/s1, Table S1: *In vivo* tolerance grading system for macroscopically evaluated signs, Table S2: Linear regression parameters of the HPLC-UV method employed for the quantitation of acetazolamide. The method was validated with respect to linearity, accuracy and reliability in the range of concentrations between 1 and 10 µg/mL.
