# Supplementary material for: Amphiphilic Acrylic Nanoparticles Containing the Poloxamer Star Bayfit® 10WF15 as Ophthalmic Drug Carriers
Source: Polymers (Basel). 2019 Jul 19;11(7):1213. doi: 10.3390/polym11071213 (PMC6680529; doi:10.3390/polym11071213)
Supplement: Supplementary file 1 [file polymers-11-01213-s001.pdf]

Supporting Information to

Amphiphilic acrylic nanoparticles containing the poloxamer star Bayfit® 10WF15 as ophthalmic drug carriers.

Miguel Gómez-Ballesteros, Vanessa Andrés-Guerrero, Francisco Jesús Parra, Jorge Marinich, Beatriz de-las-Heras, Irene Teresa Molina-Martínez, Blanca Vázquez-Lasa, Julio San Román and Rocío Herrero-Vanrell.

Supporting tables

Table S1. *In vivo* tolerance grading system for macroscopically evaluated signs.

Grade	Discomfort	Cornea	Conjunctiva	Discharge	Lids
0	No reaction	No alterations	No alterations	No discharge	No swelling
1	Blinking	Mild opacity	Mild hyperemia/mild edema	Mild discharge without moistened hair	Mild swelling
2	Enhanced blinking/intense tearing/vocalizations	Intense opacity	Intense hyperemia/intense edema/hemorrhage	Intense discharge with moistened hair	Obvious swelling

Table S2. Linear regression parameters of the HPLC-UV method employed for the quantitation of acetazolamide. The method was validated with respect to linearity, accuracy and reliability in the range of concentrations between 1 and 10 μg/mL.

	**Series 1**	**Series 2**	**Series 3**
Slope	165871	150899	163913
Intercept	3845,7	6650,9	-340,5
Coefficient of determination (r^2)	0,998	0,999	0,993
Intercept	p=0,852 N.S.	p=0,209 N.S.	p=0,973 N.S.
Slope	p<0,001 A.S.	p<0,001 A.S.	p<0,001 A.S.
